# Frailty Is Associated with Oxidative Stress in Older Patients with Type 2 Diabetes

**DOI:** 10.3390/nu13113983

**Published:** 2021-11-09

**Authors:** Blanca Alabadi, Miguel Civera, Adrián De la Rosa, Sergio Martinez-Hervas, Mari Carmen Gomez-Cabrera, José T. Real

**Affiliations:** 1 Service of Endocrinology and Nutrition, Hospital Clínico Universitario of Valencia, 46010 Valencia, Spain; balabadi@incliva.es (B.A.); mi.civeraa@comv.es (M.C.); jtreal@uv.es (J.T.R.); 2 INCLIVA Biomedical Research Institute, 46010 Valencia, Spain; adrian1031@gmail.com (A.D.l.R.); carmen.gomez@uv.es (M.C.G.-C.); 3 CIBER de Diabetes y Enfermedades Metabólicas Asociadas (CIBERDEM), ISCIII, 28029 Madrid, Spain; 4 Department of Medicine, University of Valencia, 46010 Valencia, Spain; 5 Laboratory of Exercise Physiology, Sports Science and Innovation Research Group (GICED), Unidades Tecnológicas de Santander (UTS), Bucaramanga 680006, Colombia; 6 Freshage Research Group, Department of Physiology, Faculty of Medicine, University of Valencia, 46010 Valencia, Spain; 7 CIBER de Fragilidad y Envejecimiento Saludable (CIBERFES), ISCIII, 28029 Madrid, Spain

**Keywords:** frailty, diabetes, aging, body composition, oxidative stress

## Abstract

Aging has increased the prevalence of frailty, and type 2 diabetes (T2D) has also increased in prevalence. Diabetes and oxidative stress (OS) have been shown to be related to frailty. However, the exact mechanism by which it occurs is not fully known. Our aim was to analyze body composition in community-dwelling older diabetic people treated in our center and to evaluate the possible relation between OS, frailty, and body composition. We included 100 adults older than 65 years with T2D. We found that 15% were frail and 57% were prefrail. The patients included in the nonrobust group showed increased levels of OS. Our study shows that the presence of T2D in the geriatric population is associated with a high prevalence of frailty and high OS levels, conditions that cause greater morbidity and mortality and that highlight the importance of the diagnosis of frailty in this population.

## 1. Introduction

During the last decades in industrialized countries aging has caused important changes in society. Although life expectancy has increased, there has also been an increase in the prevalence of chronic diseases such as type 2 diabetes (T2D), geriatric syndromes such as frailty, or changes in body composition that cause loss of functionality such as sarcopenia [1].

The coexistence of frailty and T2D is of special interest. Both are entities of high prevalence [2]. Furthermore, their joint presence implies a high risk of disability and a high cost for healthcare systems [3,4].

There are many metabolic changes during aging, and the increase in the percentage of body visceral fat is one of the most prominent. In contrast, lean mass (LM) and bone mass decrease [5,6]. Inflammatory state, oxidative stress (OS), mitochondrial dysfunction, malnutrition, and different energy imbalances have been implicated in the development of these changes [7], and all these processes are involved in the development of frailty and T2D [8,9]. However, the exact mechanism by which it occurs is not fully known.

**Figure 1:**
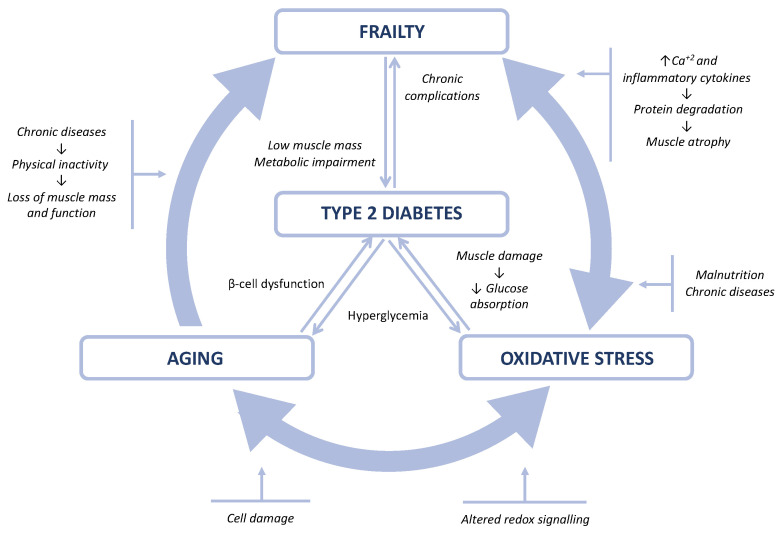
Relation between frailty, oxidative stress, aging, and type 2 diabetes mellitus.

In this sense, it has been suggested that OS could be the common link between frailty and T2D, as shown in Figure 1. Recent studies have shown that frailty is associated with a higher degree of OS [7,10,11,12]. Likewise, T2D is also associated with an increase in OS [13]. Furthermore, OS negatively affects skeletal muscle mass, which is implicated in the regulation of glucose metabolism. Finally, numerous studies showed that a low amount of LM was associated with poorer glycemic control in diabetics, a fact that highlights the importance of body composition in older adults with diabetes [14].

Therefore, the relationship between body composition and T2D is bidirectional. On one hand, because OS affects muscle, it is expected that patients with T2D will have worse body composition. As the disease progresses, musculoskeletal masses will decrease, contributing to a worse functionality compared to a healthy population. On the other hand, the changes in body composition during aging are metabolically unfavorable and increase the risk of developing diabetes [15]. Thus, it is expected that elderly with T2D present higher rates of frailty. However, the prevalence of frailty among this population varies widely [16,17,18].

Therefore, frailty screening could be useful in older adults with T2D in order to prevent and reduce the negative effects associated with the presence of frailty. In this sense, it would be of great interest to know the prevalence of frailty in this group of patients, as well as to evaluate associated factors which allow us to easily detect patients with higher risk. The objective of the present study was to analyze the prevalence of frailty in community-dwelling older diabetic people treated in our center; to analyze their body composition; and to evaluate the possible relation between OS, frailty, and body composition.

## 2. Materials and Methods

### 2.1. Participants

We included 100 elderly patients (48 men and 52 women) with T2D from the outpatient clinic of our center by consecutive sampling.

The inclusion criteria were as follows: age ≥ 65 years, diagnosis of T2D according to the ADA criteria [19], glycated hemoglobin (HbA1c) < 9%, treatment with metformin, and not being treated with allopurinol at the time of inclusion due to the possible implications of this drug on OS.

The exclusion criteria were as follows: serious chronic complications of diabetes, systemic diseases, active oncological disease, severe chronic obstructive pulmonary disease, uncontrolled hypothyroidism, cirrhosis, dementia, alterations or serious disorders of water regulation, amputations in lower limbs, or having received recent oral corticosteroid treatment for more than 30 days.

The study was approved by the Ethics Committee of our center. All patients gave their informed consent to participate in the study.

### 2.2. Clinical and Anthropometric Parameters

In the study protocol, the following clinical parameters were determined: years of disease evolution, habitual pharmacological treatment, and nutritional status using the Mini Nutritional Assessment (MNA) screening [20]. In addition, the weekly consumption of the number of servings of protein foods was determined using a food consumption frequency questionnaire.

Blood pressure was determined using a standardized procedure (mercury sphygmomanometer). Anthropometric parameters were determined using standardized procedures: weight (kg), height (m), body mass index (BMI) (kg/m^2^), waist circumference (midpoint between the last rib and iliac crest, in centimeters), calf circumference (most prominent part of the gastrocnemius muscle, in centimeters), brachial circumference (midpoint between acromion and olecranon, in centimeters), and tricipital skinfold (midline between acromion and olecranon, in millimeters).

An ergonomic, flexible, and inextensible *Cescorf* tape was used to measure the circumferences, while for the determination of the tricipital skinfold a *Holtain* LTD caliper adjusted to a pressure of 10 g/mm^2^ was used. All these measurements were made by the same researcher.

### 2.3. Body Composition and Functionality Parameters

Body composition was determined in all patients after 12 h of fasting and without having performed physical exercise in the last 8 h. A single-frequency bioelectric impedance (NutriLab, Akern, Firenze, Italy) was used, following the widely accepted methodology [21,22].

Physical performance and muscular strength were the techniques used to assess the functionality of the individuals. Physical performance was assessed using typical walking speed on a 4 m walking test, taking the smallest of three measurements and recording it in meters/second, and muscle strength was assessed using handgrip strength with a Jamar Plus+ digital dynamometer (Patterson Medical, Warrenville, IL, USA) following the recommendations of the American Society of Hand Therapists (ASHT) [23], taking the maximum of three measurements 1 min apart and recording it in kilograms.

Habitual physical activity was recorded as energy expenditure (metabolic equivalent of tasks) in 14 days (METS-min/14 days).

The relation between resistance (the opposition that the body offers to the flow of an alternating electric current, inversely proportional to the body water) and reactance (related to the capacitance properties of the cell membrane, it is the indirect reflection of the body cell mass) were used to calculate the phase angle (PA).

### 2.4. Frailty Diagnosis

Frailty diagnosis was made using the criteria proposed by Fried et al.: unintentional weight loss (10 lb in past year), self-reported exhaustion, weakness (grip strength), slow walking speed, and low physical activity. Each patient was classified as robust (when they had no altered criteria), prefrail (when they had one or two altered criteria), or frail (when they had three or more altered criteria) [24].

### 2.5. Biochemical Parameters

After 12 h of fasting, blood samples were drawn by puncture of an antecubital vein. One part of the sample was used for the determination of biochemical parameters by standardized laboratory methods, while the other was preserved in ethylenediaminetetraacetic acid (EDTA) in a BD Vacutainer tube (Becton Dickinson, Stockholm, Sweden) for the analysis of OS parameters.

Cholesterol and triglyceride levels were measured by standard enzymatic techniques [25,26]. High-density lipoprotein cholesterol (HDLc) was measured after polyanion precipitation [27], and low-density lipoprotein cholesterol (LDLc) was determined according to the Friedewald formula [28]. Glycemia was determined by enzymatic method [29] and HbA1c by high-performance liquid chromatography (HPLC) [30]. Serum albumin was detected by the bromocresol green method [31]. C-reactive protein (CRP) was measured using standard enzyme-linked immunosorbent assay (ELISA) kits (Assay test kit, St Charles, MD, USA).

### 2.6. Oxidative Stress

To extract the plasma from the blood sample, the tube was centrifuged at 1500 rpm for 15 min at 4 °C. The supernatant was stored at −20 °C until it was analyzed.

The OS parameters determined in the serum sample were malondialdehyde (MDA) and protein carbonyls. Plasma lipid peroxidation was determined following a method based on the hydrolysis of lipoperoxides in plasma and subsequent formation of an adduct between thiobarbituric acid and MDA (thiobarbituric acid—MDA2) [32]. This adduct was detected using high-performance liquid chromatography in reverse phase and quantified at 532 nm. The procedure to quantify total protein carbonyls was using the OxyBlot protein oxidation kit (Millipore Corporation, Billerica, MA, USA) and Ponceau staining followed by finding the ratio between the total density in the Oxyblot and the Ponceau.

### 2.7. Statistical Methods

The sample size was calculated using the free software G*Power. A standard alpha error of 5%, a beta error of 20%, and an effect size of 0.6 were taken into account. The minimum n obtained for the entire group was 72 patients.

Data analysis was performed using the Statistical Package for the Social Sciences (SPSS 26 for iOS, SPSS Chicago, IL, USA). For each of the variables, the values are shown as mean ± standard deviation or n (%). The p-values were bilateral, and values less than 0.05 were considered significant.

The analysis by gender was performed according to the normality of the parameters using the parametric Student’s t-test or the nonparametric Mann–Whitney U test, while one-way ANOVA test was used to analyze the differences between frailty categories. Homogeneity of the variances was tested with the Levene test, and paired comparisons were performed using Bonferroni when the homogeneity requirement was met and the Games Howell test when it was not met.

Rank biserial correlations (r), Cohen’s d values, and the partial eta squared (η^2^_p_) were used to evaluate effect size (ES) for the independent nonparametric and parametric analyses and one-way ANOVA, respectively. The effect size was interpreted using the following conventions: small effect (r ≥ 0.10, d ≥ 0.20, η^2^_p_ ≥ 0.01), medium effect (r ≥ 0.30, d ≥ 0.50, η^2^_p_ ≥ 0.06), large effect (r ≥ 0.50, d ≥ 0.80, η^2^_p_ ≥ 0.14) [33].

The multivariate correlation was studied using linear regression for frailty diagnosis, MDA, and protein carbonyls.

## 3. Results

One hundred patients were included, 48 men and 52 women. The clinical, anthropometric, body composition and functionality, biochemical, and OS variables, both in the complete cohort and by gender, are described in Table 1. According to the most relevant data, the sample is a diabetic population of long duration with a mean evolution of the disease of almost 18 years, being higher in the case of women, and with average anthropometric parameters that place them in type I obesity. We found differences between genders in some parameters, showing that women have a higher proportion of body fat (greater tricipital skinfold and fat mass index (FMI)) and a lower proportion of LM and muscle mass (lower calf circumference, fat-free mass index (FFMI), skeletal muscle mass index (SMI), and appendicular skeletal muscle mass index (ASMI)) than men. In addition, women have lower physical performance and muscle strength, as well as lower consumption of protein foods. However, there were no differences in the OS parameters.

The ES was large in all the differences obtained, except for time of T2D evolution, weekly protein rations, and total cholesterol variables, in which the ES was medium, and calf circumference variable, where small ES was obtained.

The prevalence of frailty is shown in Table 2. We found that 15% were frail and 57% were prefrail, and women were most affected.

Table 3 shows the analysis of the parameters according to frailty, dividing the group into robust, prefrail, and frail patients. Although statistical significance is not reached in both parameters, it is observed that robust patients are younger (medium effect size) and have shorter disease evolution time than those who are frail.

We found that BMI, waist circumference, and tricipital skinfold are increased in frail patients compared to robust and that mid-upper arm circumference and tricipital skinfold are increased in prefrail patients compared to robust with a medium ES. Within the body composition variables, we observed that all parameters are favorable in the case of robust patients compared to nonrobust patients, although statistically significant differences were only obtained in PA and FMI: larger PA in robust patients versus frail patients (medium ES) and lower FMI in robust patients compared to prefrail and frail (large ES). In maximum muscle strength, gait speed, and physical activity, since they are three of the five variables used for the diagnosis of frailty, we have obtained statistically significant differences between the three categories of frailty (robust versus prefrail, robust versus frail, and prefrail versus frail) with a large ES, except for physical activity, in which no differences have been obtained between robust and prefrail patients. In the same way as in the previous ones, differences are obtained between the three categories in the MNA nutritional screening score with a large ES.

With respect to parameters of OS, both are smaller in robust patients than in nonrobust; however, the distribution is different. Protein carbonyls are higher in frail patients compared to both prefrail and robust patients with a large ES. However, although MDA is higher in prefrail and frail patients compared to robust, statistical significance is only reached in prefrail patients.

Finally, a multivariate analysis was carried out to evaluate the presence of independent relation between frailty and oxidative stress (Table 4). We found that frailty was significantly related to protein carbonyls and age (Table 4A). We also evaluated the relation between OS markers (MDA and protein carbonyls) and measures of frailty (maximum muscle strength, gait speed, physical activity) as well as body composition (fat mass, muscle mass). There was not any significant relation between MDA and the parameters studied (Table 4B). However, protein carbonyl levels were related to gait speed (Table 4C).

## 4. Discussion

Population aging is a challenge for health systems [34]. Geriatric syndromes are increasingly prevalent, and even though awareness of the impact they have on quality of life and the economic burden is high, changes are still needed for the correct care of these patients, especially in those recognized as frail [24].

As there are numerous definitions and tools used to describe frailty, its prevalence varies considerably in the scientific literature. In a systematic review carried out in 2012, the prevalence varied between 4% and 59.1% in community-dwelling older adults and was limited to 9.9% in studies that used physical definitions of frailty, such as the one used in our study [35]. In 2019, Manfredi et al. obtained prevalences of 6% and 41.7% for frailty and prefrailty, respectively, in a study carried out in a population between 65 and 74 years old from 18 European countries, while for a population between 75 and 84 years old these prevalences increased to 16% and 50.5%, respectively [36]. In the FRADEA study, carried out in a Spanish population with a mean age of 79 years, the prevalence of frailty was 16.9% and that of prefrailty was 48.5% [37]. The prevalence of frailty obtained in our study, in a population with a mean age of 70.3 years, was 15%, and that of prefrailty was 57%. This prevalence is similar to those cited above, although slightly higher if we take into account that our cohort was younger. This can be explained by the presence of T2D in our study population because the chronic inflammation, the increased OS, and the insulin resistance state present in patients with diabetes cause loss of skeletal muscle mass and functionality, leading to an increase in the prevalence of frailty [38,39]. In fact, other studies that included patients with T2D showed similar results to ours [16,17,18].

Although transversal, the particularity of the present study is that all the subjects suffered T2D. There are not many longitudinal studies designed exclusively to assess differences in body composition, especially skeletal muscle loss, in diabetic versus nondiabetic older adults. Park et al. found that older adults with either undiagnosed or diagnosed T2D showed excessive loss of appendicular lean mass compared with nondiabetic subjects after six years of follow-up (loss of 0.34, 0.22, and 0.19 kg annually, respectively) [40]. These results have been subsequently repeated in a similar way in other clinical studies, with the appendicular LM generally being the main parameter affected [8,41,42]. However, we have found that the loss of LM in frail patients is less evident than the increase in fat mass, and despite the fact that a gradual trend of less lean mass is observed as frailty increases, no statistically significant differences are obtained. The loss of muscle mass is closely related to the diagnosis of frailty [43,44]. However, it has also been described in different studies how obesity can alleviate this loss of LM [45]. Furthermore, antidiabetic drugs also seem to play an important role in changes in the body composition of patients. Those most commonly used, such as insulin or metformin, affect body composition in a positive way, alleviating the previously mentioned loss of LM. Son et al. observed that intensive insulin therapy increased LM while fat mass remained stable [46]. Similar results were found by Juurinen et al. showing an increase in total body weight and LM without significant changes in fat mass [47]. Taking into account that obesity is much more present among the frail and prefrail than in the robust patients in our cohort and that all of them are treated with metformin, this could explain in part why muscle mass is not seen as diminished as expected.

We also analyzed differences between genders. It is known that the prevalence of frailty is higher in women, both in the general elderly population [16] and in the elderly population with diabetes [48]. These data have been confirmed in our study population (Table 1). The main differences resided in body composition. Men had a smaller fat compartment, reflected in the tricipital skinfold and the FMI, and a greater lean compartment, reflected in the calf circumference, the FFMI, the SMI, and the ASMI. These differences in body composition between genders are reflected in a lower PA in women and were to be expected [49,50], as was the presence of lower muscle strength and poorer physical performance in women. PA is a marker of nutritional status, prognostic of diseases and health, and indicator of cellular integrity, and it has been described to be higher in men than in women [50]. All these factors, together with the lower protein consumption and a lower score on the MNA, lead to a higher prevalence of frailty and prefrailty in women. However, these differences could also be explained by the longer time of evolution of T2D.

Another factor of interest in our study was OS. Robust patients showed lower OS status reflected by significantly lower MDA and protein carbonyl levels (Table 3). However, although MDA was higher in prefrail and frail compared to robust patients, statistical significance was only reached in prefrail patients. On the contrary, protein carbonyls were higher in frail patients compared to both prefrail and frail patients with a large ES. Furthermore, frailty and protein carbonyls showed significant independent relation in multivariate analysis. Our results are in accordance with previous studies [10,11]. It has been suggested that this increase is not due to chronological age, but rather to biological age or unsuccessful aging, that is, to frailty [7]. Recently, it has been shown that in older adults between 65 and 100 years old, the markers of OS determined in our study were related to frailty but not to age or gender [32].

The relationship between OS and frailty is complex and could be explained by different hypotheses [10]. On one hand, OS induces damage to the musculoskeletal system. The increase in intracellular calcium promotes proteasomal activity and activates muscle degradation while reactive oxygen species cause a decrease in myoblasts and apoptosis of muscle cells, leading to a loss of muscle function and strength [51]. On the other hand, OS is able to trigger an immune activation that generates oxidized cellular components. Frail patients have a weakened innate immune system and elevated levels of inflammatory cytokines which promote muscle breakdown and affect important metabolic pathways that aggravate nutritional status [52,53]. Finally, there is a higher prevalence of different pathologies that can increase OS in frail patients [54].

The obtained data are clinically relevant since it has been demonstrated that frailty and prefrailty increase the risk of cardiovascular events, hospitalization, and mortality in patients with T2D, ultimately generating a greater use of healthcare services [38,55,56]. Therefore, an early diagnosis of frailty in older adults with diabetes should be a mandatory process [57], in addition to prevention and treatment. The treatment involves addressing the sarcopenia suffered by a large part of these patients. Recently, the MID-FRAIL project has shown that resistance training combined with a nutritional program improves functionality, maximum muscle strength, and muscle power in frail and prefrail patients with T2D [58]. The importance of assessing the coexistence of these two conditions is already evident in international guidelines for diabetes, which specifically propose therapeutic goals adapted for frail patients with diabetes, such as a more relaxed target of HbA1c due to increased risk of hypoglycemia and shorter life expectancy [38].

In this sense, frailty should not be considered an irreversible and unidirectional diagnosis that inevitably leads patients who suffer it to disability. Frailty is a dynamic process, and it is possible to improve the health of these patients [59]. Gill et al. demonstrated in a cohort of 754 community-dwelling older adults that after follow-up for 54 months, more than a half of the patients made a transition between categories of frailty defined according to the Fried criteria, and although the worsening from prefrailty to frailty was more common, the condition improved in up to 23% of the patients [60]. According to our results, some of the evaluated parameters are already altered in prefrail patients (Table 3). It is interesting because these alterations could be considered as early markers for an incipient frailty process, helping to improve screening for frailty. Nevertheless, more studies are necessary to confirm our results.

However, our study has some limitations. First, we have included a limited number of subjects. Furthermore, it is a population with relatively good glycemic control and good nutritional status (reflected by serum albumin, as well as MNA nutritional screening). Thus, our cohort could not represent most of the patients with T2D. Furthermore, there is not a control group without diabetes for comparison with the results obtained. Nevertheless, the present results have important implications since one of the challenges of the current healthcare system is treating or delaying the onset of frailty in older adults. We show that circulating levels of OS markers are increased in patients with T2D and frailty. Diabetes has been previously associated with higher levels of OS. Frailty has been also associated with OS. Thus, although transversal, our study suggests that OS could be responsible for the higher prevalence of frailty in patients with T2D, suggesting one of the possible mechanisms involved in the development of frailty in these patients. Designing treatments that can prevent or reverse oxidative damage could be useful for this purpose [7]. Thus, older adults with T2D deserve special attention since this increase in OS has been shown to play an important role in the development of vascular complications [61].

## 5. Conclusions

The presence of T2D in the geriatric population is associated with a high prevalence of frailty, as well as high OS levels. Both conditions have been shown to cause greater morbidity and mortality in these patients. However, frailty status is not routinely assessed. We consider that the detection and treatment of frailty in diabetic older adults should be a priority.

## Figures and Tables

**Table 1:** Characteristics of the patients with type 2 diabetes included in the study.

	Total (*n* = 100)	Women (*n* = 52)	Men (*n* = 48)	Effect Size
Age (years)	70.3 ± 3.8	70.6 ± 3.6	70.0 ± 4.0	
Time of T2D evolution (years)	17.8 ± 10.7	20.2 ± 11.2	15.3 ± 9.7 *	0.214
Systolic blood pressure (mmHg)	131.6 ± 13.6	129.6 ± 13.9	133.9 ± 13.0	
Diastolic blood pressure (mmHg)	72.9 ± 11.7	71.5 ± 12.9	74.4 ± 10.1	
Body mass index (kg/m^2^)	30.8 ± 4.2	31.0 ± 4.4	30.5 ± 4.2	
Mid upper-arm circumference (cm)	31.5 ± 3.4	32.1 ± 3.7	30.9 ± 3.0	
Calf circumference (cm)	37.2 ± 3.1	36.7 ± 3.1	37.9 ± 3.1 *	0.387
Waist circumference (cm)	106.2 ± 14.3	104.6 ± 13.4	107.9 ± 15.3	
Tricipital skinfold (mm)	20.5 ± 8.0	25.0 ± 6.4	15.6 ± 6.6 *	0.609
Resistance (Ω)	440.1 ± 67.0	474.5 ± 60.8	403.5 ± 52.7 *	1.248
Reactance (Ω)	42.1 ± 6.2	43.1 ± 6.0	4.1 ± 6.4	
Phase angle (°)	5.5 ± 0.7	5.2 ± 0.7	5.8 ± 0.7 *	0.327
Fat mass index (kg/m^2^)	8.9 ± 3.8	10.7 ± 3.8	7.0 ± 2.7 *	1.123
Fat-free mass index (kg/m^2^)	21.7 ± 2.6	20.1 ± 1.8	23.3 ± 2.3 *	1.550
Skeletal muscle mass index (kg/m^2^)	10.1 ± 1.9	8.6 ± 1.0	11.6 ± 1.4 *	2.466
Appendicular skeletal muscle mass index (kg/m^2^)	7.9 ± 1.0	7.3 ± 0.8	8.6 ± 0.9 *	1.527
Cell mass index (kg/m^2^)	11.1 ± 2.0	10.0 ± 1.5	12.3 ± 1.8 *	1.388
Maximum muscle strength (kg)	28.6 ± 10.0	21.0 ± 4.4	36.9 ± 7.6 *	2.561
Gait speed (m/s)	0.8 ± 0.2	0.86 ± 0.2	0.71 ± 0.2 *	0.441
Physical activity (METS-min/14 days)	2996.7 ± 1905.0	2840.3 ± 1826.1	3166.1 ± 1992.3	
Weekly protein rations	10.4 ± 2.8	9.8 ± 2.5	11.1 ± 3.0 *	0.234
MNA score	26.5 ± 2.1	25.8 ± 2.2	27.3 ± 1.7 *	0.352
Glucose (mg/dL)	148.2 ± 45.7	147.1 ± 47.7	149.3 ± 43.7	
HbA1c (%)	7.4 ± 1.1	7.3 ± 1.0	7.4 ± 1.1	
Total cholesterol (mg/dL)	156.1 ± 30.2	165.7 ± 29.9	145.4 ± 27.1 *	0.711
LDL cholesterol (mg/dL)	93.0 ± 22.6	96.4 ± 23.6	89.1 ± 21.1	
HDL cholesterol (mg/dL)	47.7 ± 12.8	51.6 ± 14.4	43.4 ± 9.2 *	
Triglycerides (mg/dL)	134.5 ± 62.3	141.4 ± 57.4	126.8 ± 67.0	
Albumin (g/dL)	4.2 ± 0.2	4.2 ± 0.2	4.2 ± 0.3	
C-reactive protein	4.5 ± 8.9	4.2 ± 6.7	4.8 ± 10.9	
Malondialdehyde (µM)	6.0 ± 5.8	6.1 ± 5.6	5.8 ± 6.0	
Protein carbonyls (U.A.)	81.3 ± 20.9	84.5 ± 26.3	78.0 ± 12.9	

Data are shown as average ± standard deviation. * *p* < 0.05 between women and men. Effect size is shown for statistically significant differences.

**Table 2:** Frailty prevalence in the complete cohort of patients with type 2 diabetes and by gender.

	Total	Women	Men
Robust	28 (20.0)	7 (13.5)	21 (43.8)
Prefrail	57 (57.0)	35 (67.3)	22 (45.8)
Frail	15 (15.0)	10 (19.2)	5 (10.4)

Data are shown as *n* (%).

**Table 3:** Characteristics of the patients with type 2 diabetes according to frailty categories.

	Robust (*n* = 28)	Prefrail (*n* = 57)	Frail (*n* = 15)	Effect Size
Age (years)	69.0 ± 3.0	70.4 ± 4.2	72.1 ± 2.3 *	0.06
Time of T2D evolution (years)	15.6 ± 7.1	18.8 ± 11.2	17.6 ± 13.7	
Systolic blood pressure (mmHg)	130.2 ± 11.2	132.2 ± 14.4	132.1 ± 13.6	
Diastolic blood pressure (mmHg)	75.4 ± 8.4	70.5 ± 13.0	77.9 ± 8.7	
Body mass index (kg/m^2^)	28.6 ± 3.6	31.1 ± 4.1	32.6 ± 4.6 *	0.08
Mid-upper arm circumference (cm)	30.1 ± 1.9	31.8 ± 3.5 *	32.9 ± 4.6	
Calf circumference (cm)	37.0 ± 3.1	37.2 ± 3.2	37.5 ± 3.0	
Waist circumference (cm)	100.7 ± 16.2	106.2 ± 11.7	114.0 ± 16.8 *	0.07
Tricipital skinfold (mm)	16.6 ± 6.0	21.6 ± 8.0 *	23.1 ± 9.6 *	0.09
Resistance (Ω)	422.9 ± 46.1	446.9 ± 75.7	443.4 ± 58.1	
Reactance (Ω)	41.5 ± 5.4	43.2 ± 6.2	39.7 ± 6.4	
Phase angle (°)	5.6 ± 0.6	5.5 ± 0.7	5.1 ± 0.7 *	0.07
Fat mass index (kg/m^2^)	6.5 ± 2.3	9.7 ± 3.6 *	10.5 ± 4.7 *	0.16
Fat-free mass index (kg/m^2^)	22.1 ± 2.4	21.7 ± 3.0	21.3 ± 2.0	
Skeletal muscle mass index (kg/m^2^)	10.7 ± 1.5	9.9 ± 2.1	9.6 ± 1.5	
Appendicular skeletal muscle mass index (kg/m^2^)	8.0 ± 0.9	7.9 ± 1.2	7.8 ± 0.8	
Cell mass index (kg/m^2^)	11.5 ± 1.7	11.2 ± 2.4	10.3 ± 1.7	
Maximum muscle strength (kg)	35.1 ± 9.1	27.6 ± 9.4 *	20.9 ± 7.6 *^,^**	0.21
Gait speed (m/s)	0.68 ± 0.1	0.8 ± 0.2 *	1.0 ± 0.3 *^,^**	0.32
Physical activity (METS-min/14 days)	3979.2 ± 2179.1	2925.2 ± 1559.5	1460.4 ± 1679.2 *^,^**	0.16
Weekly protein rations	11.4 ± 2.8	10.4 ± 3.3	9.6 ± 2.6	
MNA score	27.9 ± 1.4	26.4 ± 1.8 *	24.5 ± 2.5 *^,^**	0.22
Glucose (mg/dL)	138.7 ± 34.6	156.1 ± 49.3	131.6 ± 41.9	
HbA1c (%)	7.1 ± 1.0	7.5 ± 1.1	7.4 ± 1.1	
Total cholesterol (mg/dL)	159.3 ± 32.0	155.5 ± 28.4	152.5 ± 35.9	
LDL cholesterol (mg/dL)	97.9 ± 22.7	92.7 ± 21.4	85.2 ± 26.5	
HDL cholesterol (mg/dL)	49.2 ± 11.7	46.7 ± 12.6	49.0 ± 15.9	
Triglycerides (mg/dL)	121.3 ± 68.1	144.2 ± 77.7	146.1 ± 65.3	
Albumin (g/dL)	4.2 ± 0.2	4.2 ± 0.2	4.1 ± 0.3	
C-reactive protein	4.6 ± 11.6	3.7 ± 6.6	7.8 ± 11.6	
Malondialdehyde (µM)	3.7 ± 3.2	7.3 ± 6.6 *	5.9 ± 6.2	
Protein carbonyls (U.A.)	75.1 ± 13.3	79.7 ± 22.3	99.1 ± 16.8 *^,^**	0.14

Data are shown as average ± standard deviation. * *p* < 0.05 versus robust, ** *p* < 0.05 versus prefrail. Effect size is shown for statistically significant differences.

**Table 4:** Linear regression analysis for frailty (**A**) and oxidative stress parameters (malondialdehyde (**B**) and protein carbonyls (**C**)).

A. Dependent Variable: Frailty	Unstandardized Coefficients		Standardized Coefficients		
	B	SE	β	t	Significance
(Constant)	−2.148	1.261		−1.704	0.092
Malondialdehyde (µM)	0.008	0.012	0.70	0.716	0.476
Protein carbonyls (U.A.)	0.009	0.003	0.284	2.842	**0.006**
Age (years old)	0.041	0.017	0.236	2.404	**0.018**
HbA1c (%)	0.047	0.059	0.079	0.809	0.420
**B. Dependent Variable: Malondialdehyde**	**Unstandardized** **Coefficients**		**Standardized** **Coefficients**		
**Model**	**B**	**SE**	**β**	**t**	**Significance**
1 (Constant)	5.761	4.285		1.345	0.182
Maximum muscle strength (kg)	−0.034	0.067	−0.061	−0.510	0.612
Gait speed (m/s)	1.079	3.499	0.037	0.308	0.758
Physical activity (METS-min/14 days)	0.0001	0.0003	0.037	0.350	0.727
2 (Constant)	2.800	6.037		0.464	0.644
Fat mass index (kg/m^2^)	0.155	0.232	0.102	0.667	0.506
Fat-free mass index (kg/m^2^)	0.552	0.764	0.246	0.723	0.471
Skeletal muscle mass index (kg/m^2^)	−1.014	1.169	−0.320	−0.868	0.388
**C. Dependent Variable: Protein Carbonyls**	**Unstandardized** **Coefficients**		**Standardized** **Coefficients**		
**Model**	**B**	**SE**	**β**	**t**	**Significance**
1 (Constant)	65.286	14.611		4.468	**0.0001**
Maximum muscle strength (kg)	−0.047	0.231	−0.023	−0.204	0.839
Gait speed (m/s)	26.618	11.990	0.253	2.220	**0.029**
Physical activity (METS-min/14 days)	−0.001	0.001	−0.114	−1.118	0.267
2 (Constant)	72.266	21.674		3.334	**0.001**
Fat mass index (kg/m^2^)	1.445	0.800	0.276	1.806	0.074
Fat-free mass index (kg/m^2^)	−0.873	2.690	−0.108	−0.324	0.746
Skeletal muscle mass index (kg/m^2^)	1.609	4.074	0.143	0.395	0.694

Bold indicates statistical significance.

## Data Availability

All data are available from the corresponding author upon reasonable request.

## Abbreviations

ADA	American Diabetes Association
ASHT	American Society of Hand Therapists
ASMI	Appendicular skeletal muscle mass index
BMI	Body mass index
CRP	C-reactive protein
EDTA	Ethylenediaminetetraacetic acid
ELISA	Enzyme-linked immunosorbent assay
ES	Effect size
FFMI	Fat-free mass index
FMI	Fat mass index
HbA1c	Glycated hemoglobin
HDLc	High-density lipoprotein cholesterol
HPLC	High-performance liquid chromatography
LDLc	Low-density lipoprotein cholesterol
LM	Lean mass
MDA	Malondialdehyde
METS	Metabolic equivalent of tasks
MNA	Mini Nutritional Assessment
OS	Oxidative stress
PA	Phase angle
SMI	Skeletal muscle mass index
T2D	Type 2 diabetes mellitus
